# The association of environmental house dust mite allergens and crustacean allergy: The Japan Environment and Children’s Study (JECS)

**DOI:** 10.5415/apallergy.0000000000000169

**Published:** 2025-01-08

**Authors:** Reiji Kojima, Ryoji Shinohara, Megumi Kushima, Hideki Yui, Sanae Otawa, Sayaka Horiuchi, Kunio Miyake, Hiroshi Yokomichi, Yuka Akiyama, Tadao Ooka, Zentaro Yamagata

**Affiliations:** 1 Department of Health Sciences, School of Medicine, University of Yamanashi, Chuo, Yamanashi, Japan; 2 Center for Birth Cohort Studies, University of Yamanashi, Chuo, Yamanashi, Japan; 3 Department of Epidemiology and Environmental Medicine, School of Medicine, University of Yamanashi, Chuo, Yamanashi, Japan; Nagoya City University, Nagoya, Japan; National Institute for Environmental Studies, Tsukuba, Japan; National Center for Child Health and Development, Tokyo, Japan; Hokkaido University, Sapporo, Japan; Tohoku University, Sendai, Japan; (Fukushima Medical University, Fukushima, Japan; (Chiba University, Chiba, Japan; Yokohama City University, Yokohama, Japan; University of Toyama, Toyama, Japan; Kyoto University, Kyoto, Japan; Osaka University, Suita, Japan; Hyogo Medical University, Nishinomiya, Japan; (Tottori University, Yonago, Japan; Kochi University, Nankoku, Japan; Kyushu University, Fukuoka, Japan; Kumamoto University, Kumamoto, Japan

**Keywords:** Cross-reaction, crustacean allergy, house dust mite, prevalence

## Abstract

**Background:**

The higher crustacean allergy prevalence in Asia than in Western regions may be due to a shrimp–mite cross-reaction. A high environmental house dust mite prevalence may lead to increased house dust mite sensitization and thereby increase the prevalence of crustacean allergy.

**Objective:**

To determine the association between environmental house dust mite allergens and crustacean allergy in Japanese preschool children.

**Methods:**

We used data from 4,242 mother–infant dyads who participated in the subcohort study of the Japan Environment and Children’s Study, a prospective birth cohort study. A logistic regression model was used to analyze the association between house dust mite allergens in dust at 18 months and 3 years of age and crustacean allergy at the age of 4 years.

**Results:**

The crustacean allergy prevalence was 0.4%. Greater house dust mite exposure at 18 months of age was associated with a higher prevalence of crustacean allergy, although this association was not statistically significant. However, there was no positive association between house dust mite exposure at 3 years of age and crustacean allergy.

**Conclusions:**

No association between house dust mite allergen exposure in infancy and the risk of crustacean allergy at preschool age was apparent. Follow-up studies, including investigation of tropomyosin sensitization in schoolchildren, are required.

## 1. Introduction

Shellfish include crustaceans, such as shrimp and crabs, and mollusks, such as octopus, squid, and clams [1]. Shellfish allergy is a common allergy to crustaceans, especially shrimp, often with an onset from school age [1–5] and with clinical importance as it causes anaphylaxis [1–3]. The prevalence of crustacean allergy (CA) in children is reported to be 0.06% to 2% in Western regions [5, 6] and 5.1% to 7.7% in the Asia–Pacific region [7–11], with geographical variation. In South Asia, CA has a prevalence of 5% among local adolescents (aged 14–16 years) in Singapore and the Philippines, compared with 1% among expatriates [12]. House dust mites (HDM) have been proposed as a reason for the high CA prevalence in Asia [13], and the high humidity in Asia leads to a higher HDM prevalence than that seen in Europe and the United States [13].

Tropomyosin, a major allergen contributing to shrimp allergy, is a skeletal muscle protein with 91% to 100% sequence homology between shrimp and crab [14] and approximately 80% sequence homology between shrimp and HDM [15]. Shrimp tropomyosin (Pen a 1) and HDM tropomyosin (Der p 10) may therefore be cross-allergenic, and sensitization following HDM inhalation may precede shrimp allergy, as seen in pollen–fruit allergy syndrome [1]. This hypothesis is supported by studies indicating that patients with shrimp allergy exhibit almost the same Der p 10 and Pen a 1 sensitization rates [16–18]. Additionally, shrimp sensitization was observed in approximately half of an Orthodox Jewish population that does not consume shrimp for religious reasons [19]. Several studies have reported sensitization to Pen a 1 following HDM exposure [13, 20]. However, in Iceland, an environment with negligible HDM levels, the HDM sensitization rate is 6% to 9%, and the population with HDM sensitization exhibits shrimp sensitization of 58%, suggesting a possible cross-reaction from shrimp to HDM [21].

Greater environmental HDM levels increase HDM sensitization [22, 23], and a previous cohort study reported that childhood HDM sensitization is associated with increased risk of shrimp sensitization at 8 years of age [24]. Environmental HDM allergens may therefore be associated with shrimp allergy, and regulation of environmental HDM may therefore be a preventive measure against shrimp allergy. A study from the United States, which included 500 children aged 6 to 19 years, found a significant association between cockroach allergens and shrimp sensitization, but not between shrimp sensitization and HDM allergens [25]. However, this study only investigated shrimp sensitization, and associations with shrimp allergy were not investigated. Although there is an association between shrimp allergy and shrimp sensitization [1], shrimp-specific immunoglobulin E (IgE) does not allow high diagnostic accuracy, especially in Asia [26], and the association between environmental HDM allergens and shrimp allergy therefore needs to be studied directly.

The aim of this study was to determine the prevalence of CA in Japanese children at the age of 4 years and the association between environmental HDM allergens and CA.

## 2. Methods

### 2.1. Study design and participants

Ethical approval for this study was granted by the Ministry of the Environment’s Institutional Review Board on Epidemiological Studies, Tokyo, Japan (Head of Environmental Risk Assessment Office, Environmental Health and Safety Division, Dr. Naoya Tsukamoto) on September 10, 2010 and by the ethics committees of all participating institutions (IRB number: 100910001). The study participants were the mother–child dyads participating in the Japan Environment and Children’s Study (JECS) subcohort study, the protocol and baseline participant profile of which have been published elsewhere [27, 28]. Between January 2011 and March 2014, the JECS recruited more than 100,000 pregnant women through 15 regional centers covering 19 prefectures across Japan. The JECS subcohort study randomly selected 5,017 children of the 100,302 children from the main study (approximately 5%) and collected detailed data for the subcohort using a face-to-face neuropsychiatric developmental assessment, pediatric examination, blood and urine collection, and home visits (dust collection and ambient and indoor air measurement) [29]. Written informed consent was obtained from all study participants.

We used the JECS datasets jecs-ta-20190930 (2022.11.29ver) and jecs-qa-20210401 (2023.3.16ver), which were released in October 2019 and April 2021, respectively. Of the 5,017 mother–infant dyads in the subcohort study, participants were excluded if there were missing data for HDM allergens in dust at 18 months (n = 171) and 3 years of age (n = 297), and unknown CA status at age 4 years (n = 307). We further excluded participants who had never eaten crustaceans by 4 years of age (n = 108). A total of 4,134 mother–infant dyads were included as the denominator for the CA prevalence as well as for the analysis of the association between HDM dust allergens and CA allergy.

### 2.2. Variables

#### 2.2.1. Outcome

The primary outcome was the CA prevalence at 4 years of age. CA was considered positive if both of the following criteria were met: (1) caregiver-reported allergic reactions in their children and (2) complete or partial crustacean avoidance at age 4 years. Both were collected using a self-report questionnaire. These 2 criteria were chosen based on previous studies [30].

#### 2.2.2. Exposure

The main exposure was HDM allergen in dust from the children’s mattresses, determined at 18 months and 3 years of age. HDM allergen exposure was expressed as HDM allergen per square meter of sampling surface area. Trained staff used a vacuum cleaner (Model DC61, Dyson, Japan) for 2 minutes in an area of 0.5 m^2^ to collect dust in a standardized manner. The dust was frozen until analysis. HDM allergens (*Dermatophagoides pteronyssinus* 1 [Der p 1] and *Dermatophagoides farinae* 1 [Der f 1]) were measured using enzyme-linked immunosorbent assay kits (Indoor Biotechnologies Ltd., Charlottesville, VA, USA). Dust samples with undetectable Der p 1 or Der f 1 were assigned values of half the limit of detection to calculate indoor exposure. Der p 1 and Der f 1 summed up to Der 1.

To assess HDM allergen sensitization at 2 and 4 years of age, Der p 1- and Der f 1-specific IgE blood levels were measured using densely carboxylated protein microarrays. Binding units per volume (BUe/ml) were used to express the measured allergen-specific IgE blood levels as they correlate strongly by the UniCAP system [31–33]. Samples with undetectable HDM-specific IgE were assigned values of half the limit of detection for statistical analyses.

#### 2.2.3. Potential confounders

Based on the literature review, maternal history of allergy and infantile eczema were selected as potential confounders [12].

### 2.3. Statistical analyses

The association between HDM allergens in dust and CA prevalence at age 4 years was analyzed using a logistic regression model. Odds ratios and 95% confidence intervals (CIs) were estimated. HDM allergens were divided into quartiles (Q) and the references for the analyses were assigned to the lower quartile, Q1. Only infantile eczema was included in the adjusted model because maternal history of allergy was not found to be associated with CA. HDM-specific IgE levels were summarized as median by CA status and tested with the Wilcoxon rank sum test, without any adjustment. All analyses were conducted using SAS version 9.4 (SAS Institute, Cary, NC, USA). *P* value of <0.05 was considered statistically significant.

## 3. Results

The characteristics of the participants are summarized in Table 1. Infantile eczema was present in 5.0% of children and 50.6% of the mothers had a history of allergy. The median (interquartile range) Der 1 exposure in dust samples was 71.2 (17.7–305.3) ng/m^2^ at 18 months of age and 101.3 (21.2–388.4) ng/m^2^ at 3 years of age.

**Table 1.** Characteristics of the study participants

	n = 4,242
	Number (%)
Maternal history of allergy	
Yes	2,146 (50.6)
No	2,096 (49.4)
Maternal age at pregnancy, years	
<25	399 (9.4)
≥25 and <30	1,093 (25.8)
≥30 and <35	1,524 (35.9)
≥35	1,226 (28.9)
Household income (million JPY/year)	
<4	1,476 (34.8)
≥4 and <8	2,090 (49.3)
≥8	497 (11.7)
Unknown	179 (4.2)
Mode of delivery	
Cesarean section	780 (18.4)
Vaginal	3,454 (81.6)
Birth weight, g	
<2,500	344 (8.1)
≥2,500	3,898 (91.9)
Child’s sex	
Male	2,139 (50.4)
Female	2,103 (49.6)
Older siblings	
Yes	2,352 (55.5)
No	1,890 (44.6)
Duration of exclusive breast-feeding	
<6 months	2,430 (57.6)
At least 6 months	1,788 (42.4)
Passive smoking during pregnancy	
Yes	708 (16.8)
No	3,501 (83.2)
Doctor-diagnosed allergy	
Infantile eczema	211 (5.0)
Asthma	359 (8.5)
Crustacean introduction at 1 year of age	
≤6 months	11 (0.3)
7–8 months	91 (2.2)
9–10 months	290 (7.0)
11–12 months	486 (11.7)
>13 months	57 (1.4)
Not ingested	3,219 (77.5)

CA status at age 4 years is summarized in Table 2. Among the 4,242 participants, 2.6% of participants had never eaten crustaceans, 95.0% had eaten crustaceans without allergy symptoms, 2.0% had partially eliminated them from their diet, and 0.4% had previously eaten crustaceans but now eliminated them from their diet completely. In addition, 77.2% of 1-year olds, 22.6% of 1.5-year olds, 9.5% of 2-year olds, and 3.3% of 3-year olds were reported to have never eaten crustacean (data not shown in the table). Among the 4,134 participants, there were 19 (0.5%) participants who had allergic symptoms after consuming crustaceans at age 4 years, with skin symptoms being the most common (19 participants). No participant had shock symptoms. There were 7 (0.2%) participants with crustacean sensitization and 17 (0.4%) with CA who had allergic symptoms and subsequently eliminated crustaceans from their diet.

**Table 2.** Crustacean allergy status at 4 years of age

	n = 4,242
	Number (%)
Crustacean consumption status at 4 years of age	
No avoidance (eating normally)	4,030 (95.0)
Has never eaten	108 (2.6)
Partial avoidance	86 (2.0)
Has eaten before, however, now complete avoidance	18 (0.4)
Crustacean allergy symptom at 4 years of age*	
Any symptoms	19 (0.5)
Skin symptoms	19 (0.5)
Nasal symptoms	2 (0.1)
Respiratory symptoms	2 (0.1)
Gastrointestinal symptoms	1 (0.02)
Shock symptoms	0 (0.0)
Crustacean sensitization*†	7 (0.2)
Crustacean allergy*‡	17 (0.4)

* “Has never eaten” was excluded and 4,134 participants were used as the denominator.

† Crustacean sensitization met the following criteria: abnormal blood or skin test results in the clinic or hospital.

‡ Crustacean allergy met the following 2 criteria: (1) parent-reported allergic reactions and (2) complete or partial crustacean avoidance at age 4 years.

The association between HDM allergen exposure and CA is shown in Table 3. Although a higher CA prevalence was associated with greater HDM allergen exposure at 18 months of age, this association was not statistically significant (adjusted odds ratio [aOR] Q3 vs Q1 = 1.37; 95% CI = 0.41–7.20; aOR Q4 vs Q1= 1.67; 95% CI = 0.40–7.02; *P* for trend = 0.48). However, there was no positive association between HDM exposure at 3 years of age and CA. An additional sensitivity analysis with egg allergy as the outcome showed no association between HDM allergen exposure at 18 months of age and egg allergy (Supplementary Table S1, http://links.lww.com/PA9/A44).

**Table 3.** Odds ratios of crustacean allergy in relation to Der 1 level in the mattrass

		No./total no.*	(%)	Crude OR	95% CI	Adjusted OR†	95% CI
Der 1 (ng/m^2^) at 18 months of age							
Q1	<17.7	3/1035	0.29	Ref		Ref	
Q2	≥17.7, <71.2	4/1032	0.39	1.34	0.30–6.00	1.37	0.31–6.13
Q3	≥71.2, <305.3	5/1033	0.48	1.67	0.40–7.02	1.71	0.41–7.20
Q4	≥305.3	5/1034	0.48	1.67	0.40–7.01	1.67	0.40–7.02
Der 1 (ng/m^2^) at 3 years of age							
Q1	<21.2	5/1033	0.48	Ref		Ref	
Q2	≥21.2, <101.3	5/1034	0.48	1.00	0.29–3.46	1.03	0.30–3.58
Q3	≥101.3, <388.4	3/1033	0.29	0.60	0.14–2.51	0.63	0. 51–2.65
Q4	≥388.4	4/1034	0.39	0.80	0.21–2.98	0.82	0.22–3.05

CI, confidence interval; OR, odds ratio; Q, quartile.

* “Has never eaten crustaceans” was excluded and 4,134 participants were included in the analyses.

† Adjusted for infantile eczema.

The median HDM-specific IgE levels by CA status are summarized in Table 4. Participants with CA exhibited higher levels of both Der p 1- and Der f 1-specific IgE significantly at 2 years of age, but not significantly at 4 years of age.

**Table 4.** House dust mite-specific IgE in relation to crustacean allergy

Crustacean allergy	Number*	Median (IQR)	*P* value†
Der p 1-specific IgE (BUe/ml) at 2 years of age			
Yes	17	21.8 (0.005–249.0)	0.04
No	3,944	0.005 (0.005–27.8)	
Der f 1-specific IgE (BUe/ml) at 2 years of age			
Yes	17	18.5 (0.005–243.1)	<0.01
No	3,944	0.005 (0.005–5.9)	
Der p 1-specific IgE (BUe/ml) at 4 years of age			
Yes	12	33.3(0.005–902.7)	0.14
No	3,713	0.005 (0.005–55.0)	
Der f 1-specific IgE (BUe/ml) at 4 years of age			
Yes	12	20.9 (0.005–905.6)	0.14
No	3,713	0.005 (0.005–61.0)	

IgE, immunoglobulin E; IQR, interquartile range.

* Only participants who had IgE measurement data were analyzed.

† The Wilcoxon rank sum test was used for the analysis.

## 4. Discussion

In this study, the prevalence of CA in Japanese 4-year olds was 0.4%, and participants with greater HDM allergen exposure appeared to have a higher prevalence of CA, but without statistical significance at 18 months of age. However, there was no positive association between HDM exposure at 3 years of age and CA. Participants with CA exhibited significantly higher levels of both Der p 1- and Der f 1-specific IgE than children without CA at 2 years of age, but this was not significant at 4 years of age.

In a study of approximately 100,000 Japanese nursery schoolchildren aged 6 years and younger, Noda [7] reported a CA prevalence of 0.35%. As approximately 60% of the children were aged 3 years or younger [7], CA may have been less prevalent than in our study, which included 4-year-old children. Previous studies reported CA prevalence in children under 6 years of age of 0.4% (3–4 years) in South Korea [8], 0.9% (4 years) to 1.2% (4–6 years) in Singapore [11, 12], 1.0% (under 3 years) in Taiwan [10], 1.3% (2–7 years) in Hong Kong [34], 1.7% (3–6 years) in Thailand [9], and 1.1% to 1.2% (3–5 years) in the United States [5, 6], with no large geographical variations [35]. However, geographical variations become apparent once these children reach adolescence. CA is more common in high school students, at 5.2% in adolescents (14–16 years) from Singapore and 5.1% in the Philippines, compared with 1.0% in expatriate adolescents [12], and these geographical variations may be attributed to differences in shrimp consumption or a shrimp–mite cross-reactivity. It is thought that high HDM levels in South Asia increase HDM sensitization, which may result in a higher prevalence of shrimp allergy [1].

In the present study, higher environmental HDM allergen levels at 18 months of age were associated with increased CA prevalence, but with no statistical significance. Moreover, sensitivity analysis using egg allergy, considered a negative control, was performed to ascertain whether HDM exposure specifically affects CA; this showed no association between HDM allergens and egg allergy. However, there was no positive association between HDM exposure at 3 years of age and CA. This result corresponds to the results of a previous study by Wang et al. [25] who found a significant association between cockroach allergens in dust and shrimp sensitization, but not with HDM allergens. The lack of significant associations may be due to the participants in the study being only 4 years old, a period before the onset of CA [4], and the low CA prevalence may have resulted in low statistical power. CA commonly develops in schoolchildren, which supports the hypothesis that HDM sensitization precedes the onset of CA [1]. Follow-up studies are therefore needed to investigate the prevalence of CA and the association between HDM allergens in dust and CA in school-age children.

Alternatively, the lack of a significant association between environmental HDM allergens and CA in the present study may be due to the small effect of HDM allergens in dust on tropomyosin sensitization. The amount of HDM allergens (Der p 1 or Der f 1) in dust correlates with HDM sensitization [22, 23]. However, HDM tropomyosin (Der p 10), a major cross-allergen for HDM and shrimp, is only a minor allergen among HDM allergens [36] and may therefore not induce Pen a 1 sensitization. Even in populations sensitized to HDM, less than 10% Pen a 1 sensitization was observed [37], and high environmental HDM allergen levels may not necessarily lead to sensitization toward the minor allergen [38], HDM tropomyosin Der p 10. Furthermore, geographical variations have been reported for correlations between Der p 1 and Der p 10, with correlations observed in Western studies [26], but not in Asian studies [17, 18]. Geographical variations have also been reported for tropomyosin sensitization among individuals with CA. In Western regions, the prevalence of positive Pen a 1-specific IgE in CA was as high as 70% to 89% [39, 40], whereas in Asia and other regions, including Japan, it was as low as 15.8% to 26% [18, 20], suggesting the presence of shrimp allergens other than tropomyosin [16, 18]. Recent molecular analyses have identified minor allergens in shrimp, including arginine kinase (Pen m 2), myosin light chain, and sarcoplasmic-binding protein [16, 41, 42].

In the present study, children with CA had significantly higher HDM-specific IgE levels (Der p 1 and Der f 1) at 2 years of age, which is consistent with the results of several previous studies and may be due to a correlation between Der p 1- and Der p 10-specific IgE levels [26]. However, the association was not significant at 4 years of age, which is in line with some Japanese studies reporting no correlation between Der p 1- and Der p 10-specific IgE levels [18]. As Der p 10 was not measured in the present study, it is not known whether there is a correlation between Der p 1- and Der p 10-specific IgE levels. In Asia, including Japan, HDM sensitization (Der p 1 and Der f 1) is very common [22], which may explain the discrepancy between Der p 1- and Der p 10-specific IgE levels.

The present study investigated the association between HDM allergens in dust and subsequent CA in a relatively large population. The association between environmental HDM allergens and CA is unclear, at least in preschool-aged children. The results of our study suggest that reducing environmental HDM allergens does not prevent the development of CA. However, this study has several limitations. First, tropomyosin blood levels were not measured, and future studies should investigate tropomyosin levels to determine whether HDM allergens in dust are involved in the cross-reactivity between crustacean and HDM allergens. However, the diagnostic accuracy of tropomyosin in CA is low in Asia, with a sensitivity of 34% to 37% and specificity of 80% to 80% [18, 20] and the results therefore require cautious interpretation. Second, shrimp allergy is based on parent-reported symptoms. Shrimp-specific IgE has low diagnostic accuracy [1], especially in Asia, and the food challenge test is the gold standard for shrimp allergy diagnosis. However, previous studies on the prevalence of shrimp allergy diagnosed by food challenge testing are limited [30] and the present study was a large epidemiological study, making food challenge testing difficult.

In conclusion, no association between HDM allergen exposure in infancy and the risk of CA at preschool age was apparent. The prevalence of CA in this study was only 0.4% and may therefore have low statistical power, possibly due to the children included in this study being only 4 years old and therefore younger than the expected onset of CA. Follow-up studies, including testing for tropomyosin and other allergen sensitization in schoolchildren, are required.

## Acknowledgements

The findings and conclusions of this article are the sole responsibility of the authors and do not represent the official views of the government. The authors thank all participants in the study. We also thank Melissa Leffler, MBA, Barbara Garbers, PhD, Katie Oakley, PhD, and J. Ludovic Croxford, PhD from Edanz (https://jp.edanz.com/ac) for editing drafts of this manuscript.

The JECS and the JECS subcohort study were funded by the Ministry of the Environment, Japan.

Members of the JECS Group as of 2023: Michihiro Kamijima (principal investigator, Nagoya City University, Nagoya, Japan), Shin Yamazaki (National Institute for Environmental Studies, Tsukuba, Japan), Yukihiro Ohya (National Center for Child Health and Development, Tokyo, Japan), Reiko Kishi (Hokkaido University, Sapporo, Japan), Nobuo Yaegashi (Tohoku University, Sendai, Japan), Koichi Hashimoto (Fukushima Medical University, Fukushima, Japan), Chisato Mori (Chiba University, Chiba, Japan), Shuichi Ito (Yokohama City University, Yokohama, Japan), Zentaro Yamagata (University of Yamanashi, Chuo, Japan), Hidekuni Inadera (University of Toyama, Toyama, Japan), Takeo Nakayama (Kyoto University, Kyoto, Japan), Tomotaka Sobue (Osaka University, Suita, Japan), Masayuki Shima (Hyogo Medical University, Nishinomiya, Japan), Seiji Kageyama (Tottori University, Yonago, Japan), Narufumi Suganuma (Kochi University, Nankoku, Japan), Shoichi Ohga (Kyushu University, Fukuoka, Japan), and Takahiko Katoh (Kumamoto University, Kumamoto, Japan).

## Conflicts of interest

The authors have no conflicts of interest.

## Author contributions

RK contributed to the conception and design of the study, performed the statistical analysis, and prepared the manuscript. MK, SO, H Yui, H Yokomichi, YA, TO, KM, and SH reviewed the manuscript critically. RS and ZY critically reviewed the manuscript and supervised the study. All authors read and approved the final manuscript.

## Supplementary material

Supplementary Table S1 can be found via 10.5415/apallergy.2022.12.e38.

Supplementary Table S1

Click here to view
